# Genome-wide identification and analysis of A-to-I RNA editing events in bovine by transcriptome sequencing

**DOI:** 10.1371/journal.pone.0193316

**Published:** 2018-02-22

**Authors:** Mohammad Reza Bakhtiarizadeh, Abdolreza Salehi, Rocío Melissa Rivera

**Affiliations:** 1 Department of Animal and Poultry Science, College of Aburaihan, University of Tehran, Tehran, Iran; 2 Division of Animal Sciences, University of Missouri, Columbia, MO, United States of America; Kunming University of Science and Technology, CHINA

## Abstract

RNA editing increases the diversity of the transcriptome and proteome. Adenosine-to-inosine (A-to-I) editing is the predominant type of RNA editing in mammals and it is catalyzed by the adenosine deaminases acting on RNA (*ADARs*) family. Here, we used a largescale computational analysis of transcriptomic data from brain, heart, colon, lung, spleen, kidney, testes, skeletal muscle and liver, from three adult animals in order to identify RNA editing sites in bovine. We developed a computational pipeline and used a rigorous strategy to identify novel editing sites from RNA-Seq data in the absence of corresponding DNA sequence information. Our methods take into account sequencing errors, mapping bias, as well as biological replication to reduce the probability of obtaining a false-positive result. We conducted a detailed characterization of sequence and structural features related to novel candidate sites and found 1,600 novel canonical A-to-I editing sites in the nine bovine tissues analyzed. Results show that these sites 1) occur frequently in clusters and short interspersed nuclear elements (SINE) repeats, 2) have a preference for guanines depletion/enrichment in the flanking 5′/3′ nucleotide, 3) occur less often in coding sequences than other regions of the genome, and 4) have low evolutionary conservation. Further, we found that a positive correlation exists between expression of *ADAR* family members and tissue-specific RNA editing. Most of the genes with predicted A-to-I editing in each tissue were significantly enriched in biological terms relevant to the function of the corresponding tissue. Lastly, the results highlight the importance of the RNA editome in nervous system regulation. The present study extends the list of RNA editing sites in bovine and provides pipelines that may be used to investigate the editome in other organisms.

## Introduction

The biology of the mammalian transcriptome is far more complex than once thought. Previous studies have shed light on the dynamic nature of the mammalian transcriptome, where different molecular processes interact to fine-tune gene expression [1]. For instance, large-scale projects based on high throughput cDNA sequencing technology (RNA-Seq), such as ENCODE [2] and GENCODE [3] have clearly shown that RNA transcripts undergo a host of diverse processing mechanisms. One such mechanism is RNA editing, which is defined as any post-transcriptional or co-transcriptional mechanism that alters the nucleotide composition of a transcript. Therefore, this phenomenon leads to differences between the final transcript sequence and the DNA region it was transcribed from [4]. Since its discovery in 1986 in trypanosomes [5], RNA editing has been reported to occur in a broad range of species ranging from bacteria [6] to mammals [7, 8]. As inosines are read as guanosine by the translation and splicing machineries [4, 9], RNA editing can influence alternative splicing [10], recoding of open reading frames [4] and can affect miRNA-regulated post-transcriptional gene silencing [11]. RNA editing plays vital roles in the development and maintenance of the metazoan nervous system [12], marking RNAs for degradation, modulating nuclear retention of RNAs [9] and when deregulated, this mechanism is associated with various diseases [13] and cancers [14].

There are two known types of RNA editing in mammals namely, cytosine-to-uracil (C-to-U) and adenosine-to-inosine (A-to-I) [4]. C-to-U editing is catalyzed by the apolipoprotein B mRNA editing enzyme, catalytic polypeptide-like (*APOBEC*) family [15], while A-to-I editing is catalyzed by the adenosine deaminases acting on RNA (*ADARs*) family. The latter is the predominant type of RNA editing in mammals [9]. *ADAR* enzymes bind to double-stranded RNAs (dsRNAs) through their double-stranded RNA-binding domains and deaminate adenosine to inosine. Therefore, RNA editing preferentially occurs within inverted repeat sequences such as LINE (long interspersed nuclear elements) and SINE (short interspersed nuclear elements) retrotransposons (like the primate Alu repeats), because of the dsRNA structures formed by these sequences. For instance, it has been demonstrated that most of the A-to-I editing sites in the human transcriptome are clustered within Alu repeats, which mostly reside in introns and UTRs regions of genes [4].

Novel RNA-editing sites can be discovered by direct comparison between cDNA sequences and their corresponding genomic position. Several recent next generation sequencing (NGS) based studies, have reported RNA editing sites in different vertebrates including human [8, 12, 14], mouse [16], pig [17], chicken [18] and bovine [19]. There are several challenges for identifying RNA editome using RNA-Seq data including the discrimination of true RNA editing sites from single-nucleotide polymorphisms (SNPs), somatic mutations, systematic sequencing errors and mapping errors [20]. Recently, different bioinformatics methods have been developed to discover RNA editing events by comparing RNA and DNA sequencing data collected from single individuals. As RNA-Seq and DNA-Seq datasets are not always available for the same individual, different methods have been developed to identify RNA editing sites using RNA-Seq data alone [8, 12–14, 19–25]. These methods have allowed the identification of more than 1.3 million potential RNA editing sites in human and more than 7,000 for the mouse [16]. Contrary to human and mouse, only two very recent studies have provided information about RNA editing in bovine, the first study identified 671 putative A-to-I editing sites in four fetal tissues [26] while the second study focused on one tissue (brain) to identify RNA-sequencing reads containing clusters of editing sites in 21 diverse organisms including bovine [27]. As RNA editing is developmental stage specific [28] as well as tissue-specific [29], much more work is required in order to identify the extent of this post-transcriptional mechanism in this species. The comprehensive documentation of the bovine RNA editome will provide a valuable resource for the characterization of cellular and physiologic outcomes involving this modification in this agriculturally important species.

In this study, we used a largescale computational analysis of RNA-Seq data across brain, heart, colon, lung, spleen, kidney, testes, skeletal muscle and liver, from three adult animals. We developed a computational pipeline and used a rigorous strategy in order to identify novel RNA editing sites, taking into account sequencing errors, mapping bias (misalignments around repetitive regions and splice junctions), as well as biological replicates, to control the false positive rate. We also conducted a detailed characterization of sequence and structural features related to novel candidate A-to-I editing sites and found 1,600 novel canonical A-to-I editing sites. Most of the genes with predicted A-to-I editing were significantly enriched in biological terms relevant to the function of the corresponding tissue. In addition, we found frequent occurrence in clusters and in SINE repeats, preference for strong guanines depletion/enrichment in the nucleotide 5′/3′ to the edited A-to-I sites, lower editing sites in coding sequences than other regions, low number of evolutionary conserved sites, concordance of *ADARs* expression in different tissues with editing levels and tissue specificity of editing sites.

## Material and methods

### Datasets

To identify the RNA editome in the bovine transcriptome, we retrieved 27 publicly available paired-end strand-specific RNA-Seq samples (Gene Expression Omnibus database, accession number GSE41637). The libraries were developed from three adult animals and each contained reads from nine tissues, namely brain, heart, colon, lung, spleen, kidney, testes, skeletal muscle and liver. This dataset [30] was generated using the Illumina HiSeq 2000 platform. In this dataset, only the samples of one animal were sequenced at 76 bp read length and at high coverage (average = 111.2 million reads per sample) while the samples of the other animals had moderate coverage (average of 28.5 million reads per sample with 36 bp read length).

### Pipeline for identifying novel RNA editing sites

**Fig 1:**
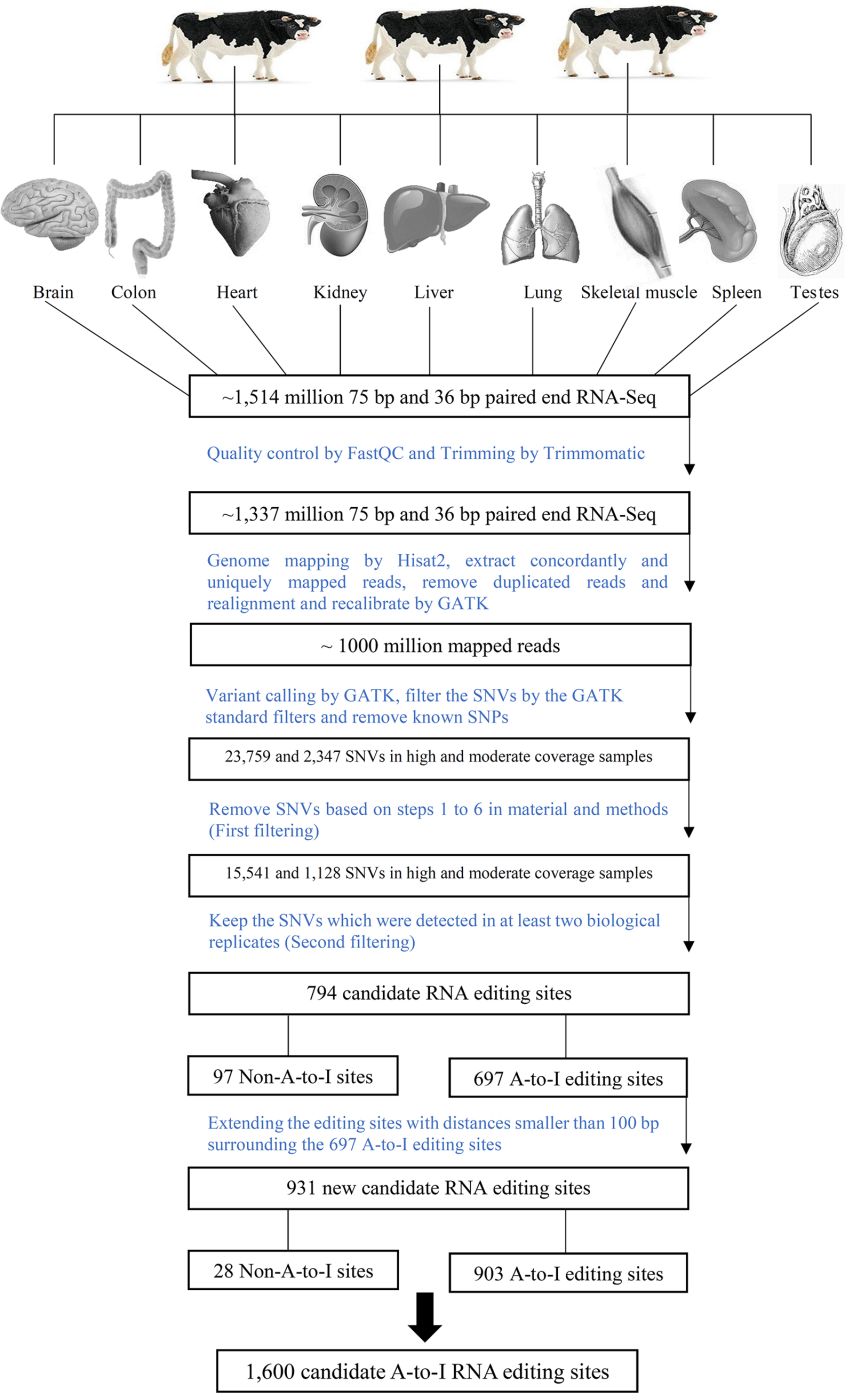
Summary of our pipeline for identifying RNA-editing sites.

Fig 1 shows an overview of the steps of our computational analysis pipeline for identifying the novel RNA editing sites of each tissue. The pipeline minimizes false positives and maximizes the prediction of true novel RNA editing as follows.

#### Quality control and read mapping

Raw read quality check was performed with FastQC v0.11.5 (http://www.bioinformatics.babraham.ac.uk/projects/fastqc/). Sequencer adapter removal and quality trimming was done with Trimmomatic [31] (parameters of Trailing 20, Maxinfo 60:0.90 and minimum length 60 for 76 bp; and Trailing 20, Maxinfo 30:0.90 and Minimum length 30 for 36 bp datasets). The trimmed reads were aligned to the Ensembl UMD3.1 bovine reference genome using Hisat2 version 2.0.5 [32]. A list of exon-exon junctions extracted from the known gene model annotation (Ensembl release 84) was used to guide the read mapping. Notable features of the Hisat2 program are that, 1) it prevents reads from being aligned to pseudogenes, which results in improved alignment accuracy [32] and 2) it is more efficient at providing editing prediction from RNA-Seq data than other programs [33]. We considered only uniquely and concordantly paired-end mapped reads, to reduce the potential bias caused by short read alignment. Also, in order to avoid potential PCR or sequencing optical artefacts influencing editing frequencies, duplicated reads that mapped to the same location were removed by MarkDuplicates tool from Picard (http://picard.sourceforge.net/), except those with the highest mapping quality score [34]. The remaining reads were locally realigned (using GATK tool) around putative insertions and deletions using Ensembl bovine SNP database version 146. Then, the base quality values were recalibrated by GATK (version 3.5, https://www.broadinstitute.org/gatk) tool.

#### Identification of candidate RNA editing

RNA editing is defined as a single nucleotide base change between DNA and RNA. RNA-Seq single nucleotide variants (SNVs) calling was performed with HaplotypeCaller [35] using GATK tool on each sample with a stand_call_conf and stand_emit_conf value of 30 and mbq of 25. Initially identified SNVs were removed from further analysis if they corresponded to known bovine SNPs found in Ensembl bovine SNP database version 146. Then, the SNVs were filtered by the GATK standard filters (HomopolymerRun >5, Total depth of coverage <10, RMSMappingQuality <40, QualitybyDepth < 2, MappingQualityRankSum < -12.5 and ReadPosRankSum <-8). These filtering parameters were used to eliminate: 1) sites with less than 10 supporting reads, 2) variant distance bias, 3) mapping quality bias, and 4) homopolymer bias [36]. The remaining SNVs were further filtered by several quality-aware steps. First, we discarded the sites with more than one nonreference type and the sites that were homozygous for the alternative allele. Second, the read coverage was restricted to at least 10 reads with at least three reads supporting the SNV site and only those sites, which were present in at least 10% of the reads, were kept for further analysis. Further, the SNVs sites with extreme degree of variation (100%) were removed under the assumption that 100% editing efficiency is unrealistic. Third, SNVs that occurred in regions with bidirectional transcription (sense and antisense gene pairs) were removed. Fourth, simple sequence repeats patterns (SSR [i.e. motifs ranging in length from 1 to 8 nucleotides with a minimum length of pattern of 6 bases]) were identified using GMATo software [37] and removed from further analysis. SNVs located in SSR regions were considered as biased with an offset of ±3 bases. Fifth, to exclude potential false positives resulting from poor mapping of reads at splice junctions, all SNVs located within 5 bp intronic flanking region were discarded. Sixth, to ensure that the reads supporting the SNVs were uniquely aligned, we filtered out SNVs in paralogs or repetitive regions by retrieving and aligning 100 bp of flanking sequence (50 upstream and 50 downstream of the SNV) using BLAT [38]. Finally, since RNA editing is tissue-specific and should be similarly present in animals within a species, while low frequency SNPs should not, the pipeline considered the number of times that the SNVs were detected in more than one individual to output a final set of predicted editing sites. Hence, all SNVs detected in at least two of the three individual bovines were considered candidate RNA editing sites, thus minimizing false positive results.

#### Annotation of the candidate RNA editing sites and enrichment analysis

As dsRNAs produced from inverted retrotransposon repeats such as LINE and SINE families are important *ADAR* substrate RNAs [4]. We investigated whether candidate RNA editing sites were enriched in these regions. For this, all interspersed repeats sequences (UCSC database [http://genome.ucsc.edu]) detected by RepeatMasker program [39] were screened to identify the candidate RNA editing sites.

The functional annotation and genomic location of each candidate RNA editing in each gene was identified using SnpEff (v 4.3) [40]. In order to identify the biological functions associated with edited genes, the enriched gene ontology (GO) terms (BP, biological process) were investigated using Enrichr web-application (threshold false discovery rate (FDR)< = 0.05) [41].

### EST analysis

We used the public bovine EST sequences (ftp://ftp.ncbi.nih.gov/repository/UniGene/) to investigate whether the editing events identified by our pipeline were also present in these sequences. For this, 50 bp upstream and 50 bp downstream flanking regions were extracted and queried against the bovine EST sequences using BLAST. Alignments with e-values less than 10^−5^ were considered as significant and counted.

### Conservation analysis

We performed cross-species transcriptome comparisons between editing sites identified in this study and those reported in human in order to identify highly conserved A-to-I editing events. Fifty base pairs flanking regions of the bovine candidate sites were BLAST against 50 bp flanking regions of the known human sites. The known editing sites in human (hg19) were retrieved from the RADAR database (http://rnaedit.com). The hits with e-values <0.001, >0.85% identity and >50 bp alignment length were considered as conserved editing sites.

### Analysis of neighbor preferences

Previous studies reported that ADAR enzymes have sequence preference for the edited site neighbor nucleotide [4]. Base preference around the identified candidate RNA editing sites was investigated by extracting 10 bp upstream and 10 bp downstream of the edited sites. Sequence logo was then generated by WebLogo software [42].

### Gene expression quantification

The clean reads samples were loaded into Salmon (0.8.1) pseudo-alignment transcript quantification [43], with sequence and GC bias correction enabled and using k = 25 for indexing, to quantify transcript-level abundances. Transcripts per kilobase million (TPM), which normalizes for transcript length and sequencing depth was used as an estimate for relative expression level. To improve reliability of quantification, the transcript-level quantifications were merged to the gene level by summing the corresponding transcript-level TPM estimates.

### Statistical analysis

Statistical significance for differences between tissue means for editing ratio were assessed by Student's unpaired t-test and were implemented with R. FDR corrected P-values were used to control false positives resulting from multiple testing (corrected P-value < 0.05).

## Results

### Identification of RNA editing in bovine

Computational identification of RNA editing is dependent on the stringency levels used to remove false positives. This is especially true when solely using transcriptome data [44]. To capture and characterize the complexity of the editome in the bovine transcriptome, we exploited existing strand-specific RNA-Seq datasets from nine tissues of three individual animals. We designed a computational approach that implements multiple filters with stringent thresholds (Fig 1).

After quality trimming, >88% of the 1,337 million reads contained in those datasets aligned to the bovine reference genome (UMD3.1) and ~76% (i.e. 1 billion reads) were uniquely aligned (S1 File). Initial analysis of the high coverage samples identified 809,332 SNVs. The average number of variants was 195,574 (range in 9 tissues = 53,577 to 299,526). A total of 247,251 SNVs were detected in the 18 datasets of moderate coverage. The average number of SNV per tissue was 39,701 (range in 18 tissue samples = 14,251 to 58,300). In the high coverage group, 23% of variants in all tissues were A-to-I while in the moderate coverage group this range was 19%. This initial SNV list was further subjected to stringent filtering to control for false-positives. This step led to the identification of 15,541 SNVs in the high coverage sample and 1,128 SNVs in the moderate coverage samples of which 0.90 and 0.69 were A-to-I type, respectively (Fig 1).

**Fig 2:**
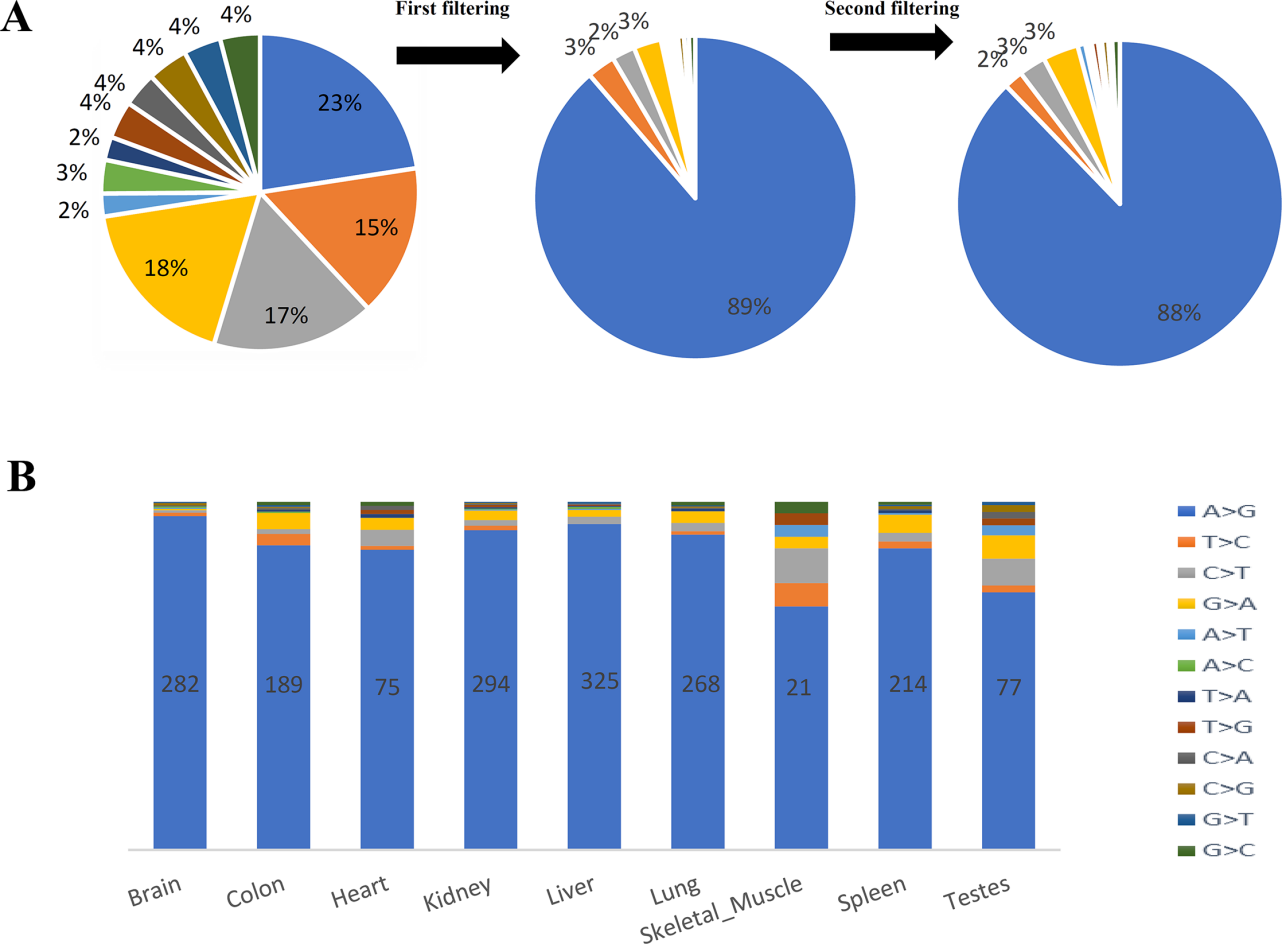
Distribution of the identified RNA editing sites using RNA-Seq data in bovine. A) Distribution of all 12 mismatch types in bovine transcriptome throughout the different steps of pipeline. In the first filtering step, we removed known SNPs, low-quality base calling, homozygous sites, sites with low read coverage, lowly or highly edited sites, sites with strand bias, variant distance bias, mapping quality bias, homopolymer bias, also variations which were located in bidirectional, exon-intron boundaries and paralogs or repetitive regions. In the second filtering step, we kept the sites which were observed in at least two biological replicates of cattle. B) The final distribution of all 12 mismatch types in different tissues. The identified variants were required to be present in least two or three biological replicates.

A second filtering step was used to obtain a comprehensive map of potentially editable sites in the bovine transcriptome and identified 794 common SNVs in at least two bulls of which 88% were considered to be potential A-to-I RNA editing sites (Fig 2A, S2 File). These were located in unique genomic positions and were not close to any splice junction, bidirectional transcription or low complexity regions (such as SSRs). Of these, ~88% (n = 697) corresponded to A-to-I differences and ~3% (21 sites) were C-to-U (Fig 2A, S2 File). We focused further analysis on the canonical A-to-I editing sites.

### RNA editing distribution in different tissues

**Fig 3:**
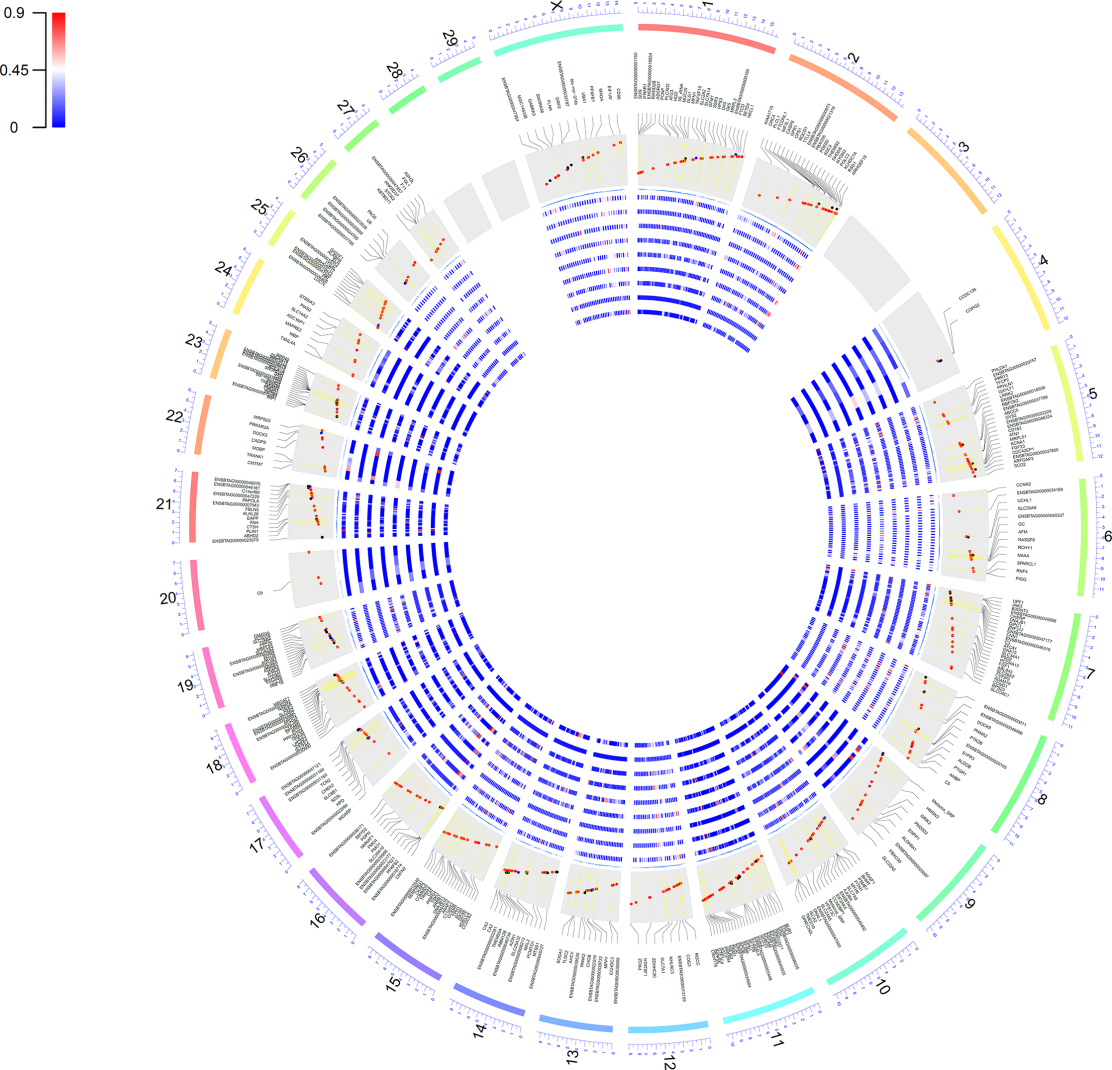
Distribution of putative RNA editing sites across bovine chromosomes. The bovine genome is shown as a circle. For each chromosome, the position of the editing sites along with their average editing ratio (heatmap) is shown in different tissues. The red, blue and black dots in the inner light gray circle indicate the A-to-I, C-to-U and other non-canonical editing sites, respectively. Also, the vertical yellow lines in the inner light gray circle indicate the position of inverted repeats. Tissues are shown in concentric circles and ordered as follow from the outside: brain, colon, heart, kidney, liver, lung, skeletal muscle, spleen and testes.

**Fig 4:**
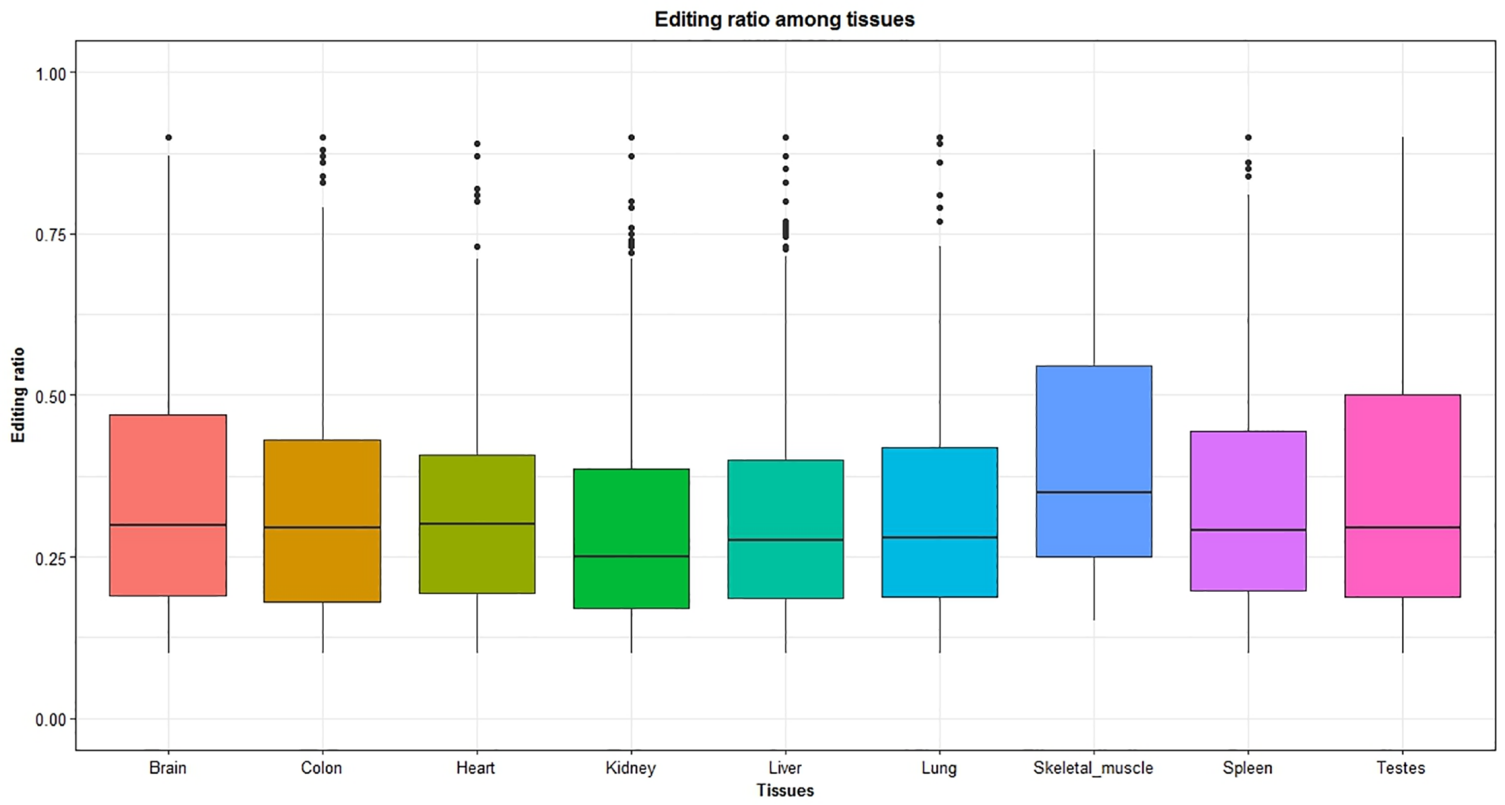
Distribution of RNA editing ratio across bovine tissues. The Y-axis shows the editing ratio of edited genes and the X-axis shows the different tissues. The middle line in each box indicate the mean value of editing ratio in each tissue.

The RNA editome (A-to-I events) varied per tissue. The tissue with the highest number of edited sites was the liver (n = 325; Fig 2B) while the lowest number of sites were detected in the muscle (n = 21; Fig 2B). The distribution of RNA editing ratio, however, was similar across tissues (Figs 3 and 4, S4 File). Tissue profiling identified the brain as the tissue with the highest editing ratio with 23% of edited sites having an editing ratio >0.50%. However, the mean editing ratio were not significantly different (corrected P-value <0.05) among tissues, except between brain and kidney (S4 File). Specifically, for A-to-I sites, we identified 320 genes harboring at least one site within the gene or their 5 kb flanking regions. The number of edited sites detected in brain, colon, heart, kidney, liver, lung, skeletal muscle, spleen and testes was 146, 97, 41, 153, 156, 136, 13, 112 and 45, respectively. Fig 3 shows a circos plot depicting the landscape of all RNA-DNA mismatches identified in this study across bovine chromosomes. A comprehensive list of all the edited genes in different tissues with number of editing sites in each gene is presented in S5 File.

#### Whole genome distribution of editing sites

**Fig 5:**
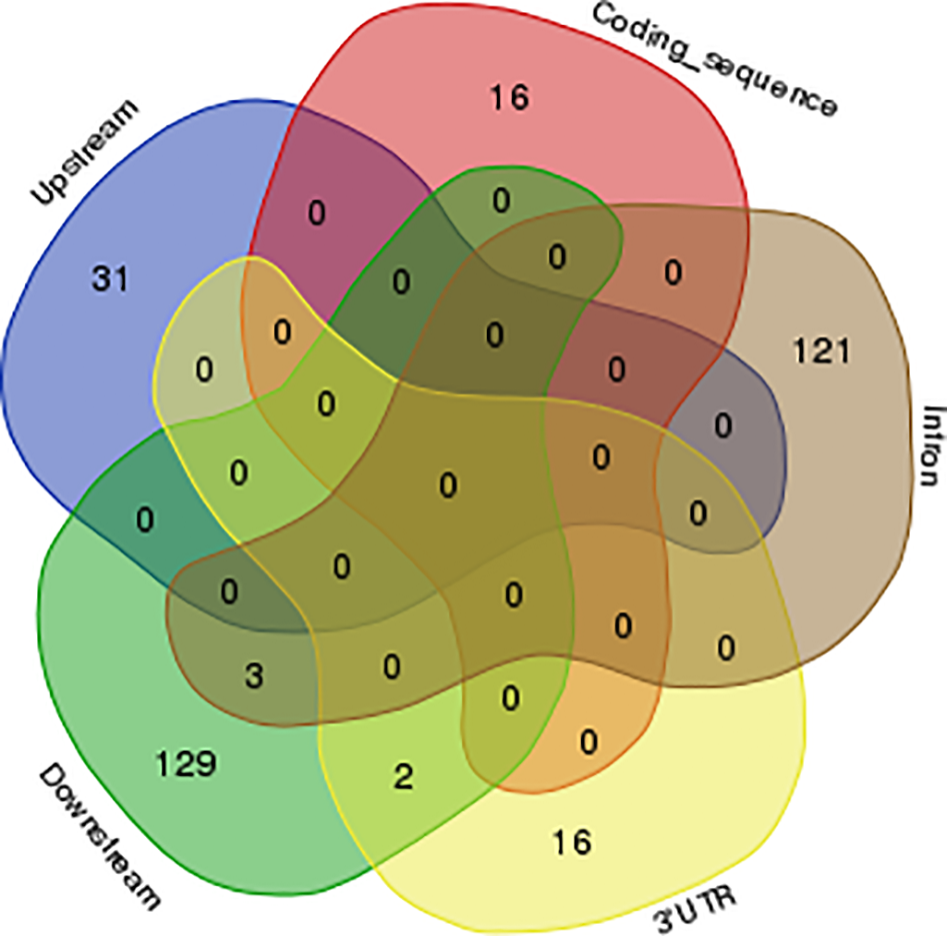
Venn diagram of edited genes showing the genomic location of their editing sites. The figure shows that simultaneous editing of genes in more than one position in gene is rare as there are only five genes which were edited in two positions.

**Fig 6:**
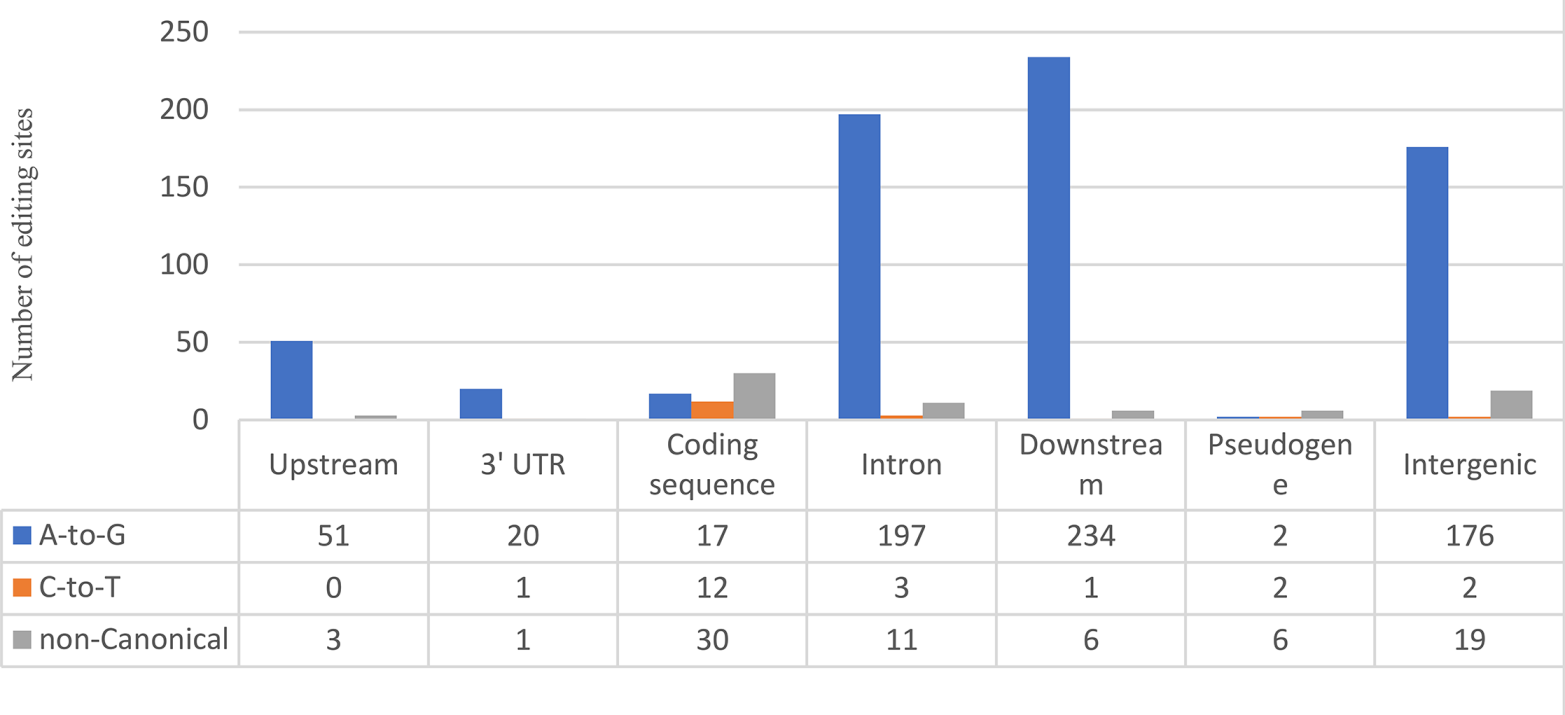
Distribution of genomic location of the identified editing sites. The Y-axis shows the number of editing sites and the X-axis shows the position. Blue, orange and gray colors indicate the A-to-I (or A-to-G), C-to-U and non-canonical editing sites, respectively.

Next, we assessed the genomic locations of A-to-I editing sites in the bovine genome. These sites were not evenly distributed across the genome. For example, transcripts from genes in chromosomes 3, 28 and 29 were not observed to be edited in the dataset we analyzed while several (n = 23) of those in chromosome 11 were (Fig 3). All together 678 of 697 edited events were observed in non-coding regions such as introns, intergenic and untranslated regions. Of those, 285 intergenic associated sites were located within 5 kb of annotated genes and some may represent extended 3′ UTR regions. Furthermore, in agreement with previous study, most of the edited genes were edited only in one of the genic regions such as intron, coding sequence (CDS) or 3′ UTR. The results of this analysis showed that only *AJUBA* and *EAPP* were edited simultaneously in the 3’UTR and the downstream. In addition, *CD99*, *GK5* and ENSBTAG00000038619 were edited simultaneously in an intron and downstream regions (S3 File, Fig 5) [45]. Distribution of genomic location of A-to-I, C-to-U and non-canonical editing sites are shown in Fig 6.

Only 17 A-to-I editing sites were located in CDSs, of which 15 editing sites changed the amino acids of 14 genes (either non-synonymous or missence; S3 File, Table 1). Of those, the brain had the largest number of CDS edited genes namely, *CADPS*, *CYFIP2*, *GABRA3*, *GIPC1*, *GRIK2*, *KCNA1*, *LEMD2*, *TMEM63B*, *SON* and ENSBTAG00000047954 while skeletal muscle had none (S5 File).

**Table 1:** List of edited genes with non-synonymous effect.

Positon	Gene	Effect of editing	Positon	Gene	Effect of editing
14:63799763	*AZIN1*	Aspargine to Aspartic acid	23:7857711	*LEMD2*	Serine to Glycine
22:39005364	*CADPS*	Glutamic acid to Glycine	23:17795420	*TMEM63B*	Glutamine to Arginine
12:15671536	*COG3*	Isoleucine to Valin	1: 1258231	*SON*	Arginine to Glycine
7:71024261	*CYFIP2*	Lysine to Glutamic acid	1:1258705	*SON*	Threonin to Alanin
X:34866869	*GABRA3*	Aspargine to Aspartic acid	13:43765915	*ENSBTAG00000039722*	Lysine to Arginine
7:12391977	*GIPC1*	Threonine to alanine	8:40110977	*ENSBTAG00000046486*	Lysine to Glutamic acid
9:48600848	*GRIK2*	Glutamine to Arginine	X:18885623	*ENSBTAG00000047954*	Arginine to Glycine
5:105663515	*KCNA1*	Isoleucine to Valin			

To further evaluate whether the identified A-to-I editing sites in our study are potential editing events, we investigated different sequence and structural features that have been shown to be consistent with the known properties of A-to-I editing.

### Tissue-specific A-to-I editing sites

We first focused on tissue-specific A-to-I sites. We found 107, 6, 12, 34, 103, 15, 2, 7 and 3 tissue-specific sites, which were dispersed among 53, 5, 6, 12, 50, 9, 0, 4 and 2 genes in brain, colon, heart, kidney, liver, lung, skeletal muscle, spleen and testes, respectively. To investigate whether tissue specificity of edited genes was involved in functional processes relevant to that tissue, functional enrichment analysis was performed for each tissue. Results showed that these genes were significantly enriched in various biological processes associated with function of the associated tissue. For example, some of the significantly enriched biological processes terms were included “regulation of short-term neuronal synaptic plasticity” in brain (adjusted P-value = 0.05), “striated muscle contraction” in heart (adjusted P-value = 0.01), “kidney development” in kidney (adjusted P-value = 0.03), “positive regulation of lipid storage” in liver (adjusted P-value = 0.005) and “respiratory gaseous exchange” in lung (adjusted P-value = 0.02). The complete list of enrichment analysis of tissue-specific edited genes in each tissue along with the distribution of tissue-specific editing sites and their editing ratio are provided in S4 File.

We also analyzed the number of A-to-I editing sites that were shared among all the tissues. Results showed that the most commonly edited genes were *GTF3C4* and *C4A*, which were edited in at least eight tissues, 23 genes were found to be edited in at least seven tissues, and 72 were in common in at least five tissues. In addition, we found that the lung and kidney shared 163 sites (S2 File). These findings indicated that the recurrence of editing sites across tissues was low.

### ADARs expression and RNA editing levels

In order to explain differential or tissue-specific RNA editing, we first determined transcript abundance across tissues for genes that were edited in a tissue-specific manner. The rationale behind this analysis was to investigate whether tissue-specific RNA editing is correlated with the sole expression of those transcript in the tissue in which editing was observed. Our results show that tissue-specific edited genes were expressed in at least four of the nine somatic tissues examined (S6 File). Moreover, tissue-specific edited genes in lung and spleen were expressed in all other tissues. Also, more than 94% of tissue-specific edited genes in brain were expressed in at least eight other tissues.

**Fig 7:**
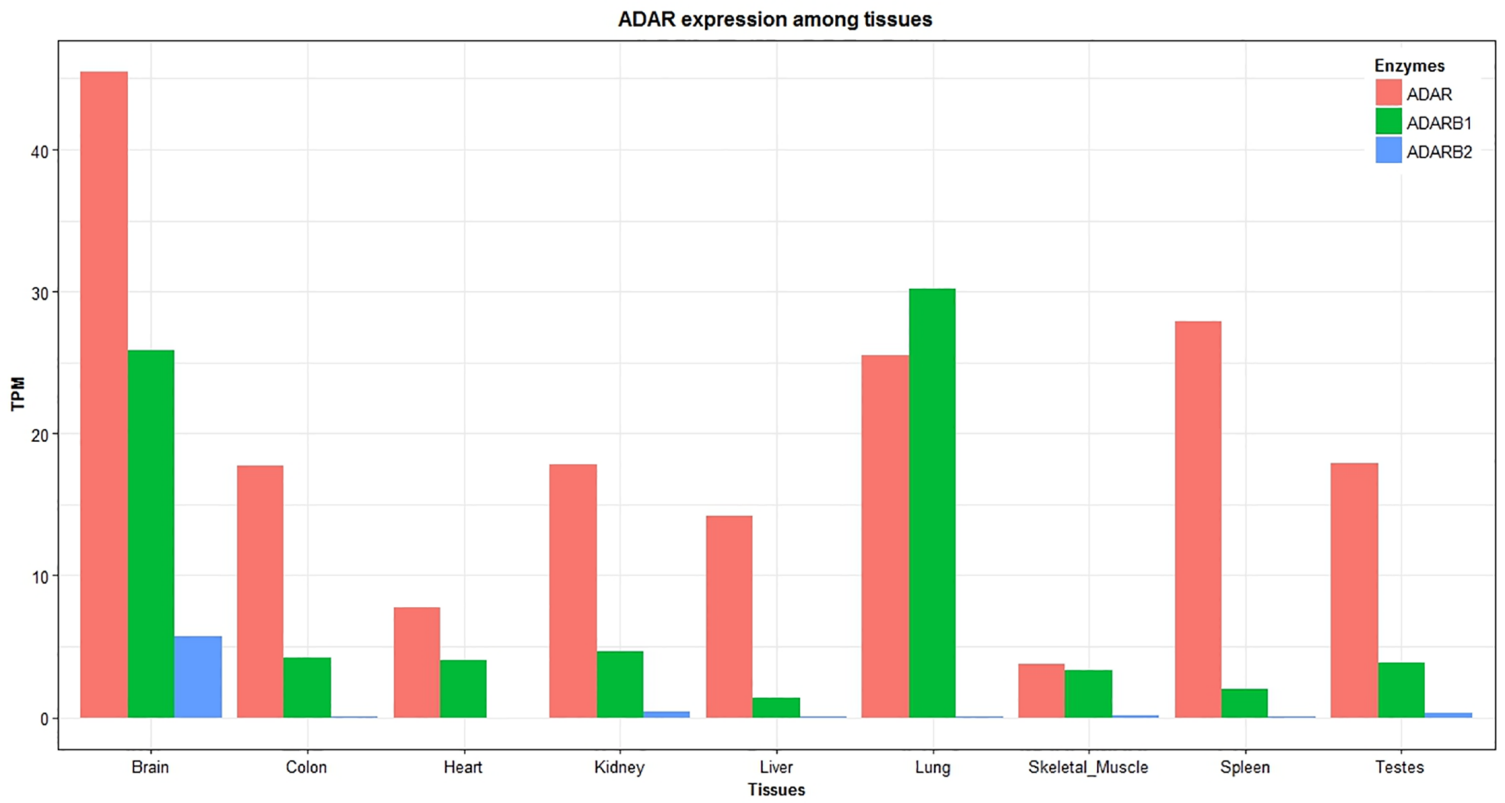
*ADAR* enzymes expression across bovine tissues. The Y-axis shows the gene expression of *ADAR* enzymes in transcripts per kilobase million (TPM) and the X-axis shows the different tissues.

Next, we analyzed if the editing pattern observed is related to the tissue-specific expression levels of *ADAR* enzymes, namely *ADAR* (or *ADAR1*), *ADARB1* (or *ADAR2*), and *ADARB2* (or *ADAR3*). Our results showed that *ADAR* had highest expression in the brain, which is consistent with higher number of tissue-specific RNA editing in this tissue. Interestingly, *ADAR* expression was the lowest in heart and skeletal muscle, which is in accordance with the lowest number of editing sites in these tissues (Fig 7). As shown in Fig 7, there is a relative relationship between *ADAR* expression and RNA editing pattern in different tissues. Overall, we found a trend for a positive correlation (Pearson correlation = 0.59, P-value = 0.1) between expression of *ADAR* family members and tissue-specific RNA editing.

### Enrichment of editing sites in inverted repeat sequences

We analyzed the enrichment of A-to-I editing sites in based on genomic context. Results showed that out of 697 A-to-I editing sites, 588 sites were found in repetitive regions. Of the 588, 535 were enriched in SINE regions (specifically in the *Bov-tA* SINE family [n = 406]), 41 in LINEs and 12 were presented in other inverted repeat regions such as DNA transposons. In contrast, only 10 of 97 non-A-to-I sites were observed in these regions (Fig 3, S3 File).

### Motif sequence

**Fig 8:**
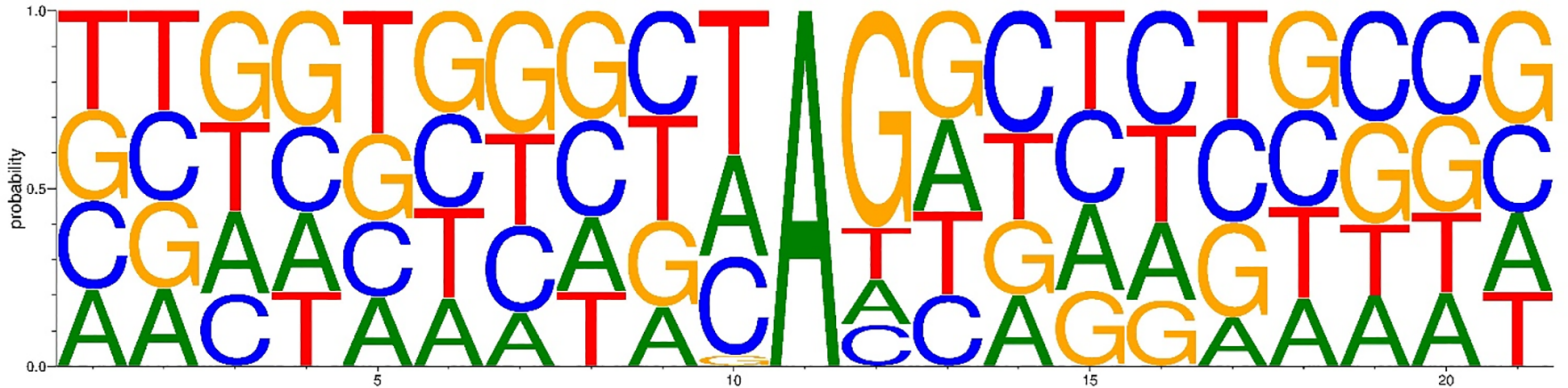
Neighbor sequence preferences of nucleotides for A-to-I editing sites (the A nucleotide at position 0).

We queried whether sequence could affect editing events. Our results show that the nucleotides 5′ and 3′ to the editing sites had a strong preference for T and G enrichment, respectively (Fig 8).

### Conservation analysis

To examine whether the editing sites we identified in bovine could also be detected in human orthologous regions, we performed cross-species comparisons between the bovine and human editomes. After applying stringent thresholds on the results (e-values <0.001, identity >0.85% and alignment length >50 bp), we observed 16 conserved A-to-I editing sites in 12 protein coding genes namely, *TMEM63B*, *SON*, *KCNA1*, *GRIK2*, *GABRA3*, *FLNA*, *COG3*, *CADPS*, *AZIN1*, *GIPC1*, *DDX6* and *ADCYAP1* (Table 2 and S3 File).

**Table 2:** Conserved A-to-I editing sites between bovine and human.

Chr:Pos	Annotation_Bovine	Annotation_Human	Tissue
5:105663515	*KCNA1*-Non_Synonymous	*KCNA1*	Brain
5:26529688	Intergenic	Intergenic	Kidney, Spleen
5:76189492	Intergenic	*ELFN2*	Brain
7:12391977	*GIPC1*-Non_Synonymous	*GIPC1*	Brain, Lung
9:2105925	Pseudogene	*uc001dua*.*2*	Lung, Skeletal muscle
9:48600848	*GRIK2*-Non_Synonymous	*GRIK2*	Brain
10:42671350	Pseudogene	*PMS2*	Heart, Lung, Skeletal muscle, Testes
10:42671603	Pseudogene	Intergenic	Colon, Kidney, Spleen
11:258705	*SON*-Non_Synonymous	*SON*	Brain, Lung
12:15671536	*COG3*-Non_Synonymous	*COG3*	All tissues except brain and skeletal muscle
12:62481777	Intergenic	*CD226*	Brain
14:63799763	*AZIN1*-Non_Synonymous	*AZIN1*	Colon, Kidney, Liver, Lung, Spleen
15:29888325	*DDX6*-3′UTR	*DDX6*	Colon, Kidney, Liver, Lung, Spleen
22:39005364	*CADPS*-Non_Synonymous	*CADPS*	Brain
23:17795420	*TMEM63B*-Non_Synonymous	*TMEM63B*	Brain
24:36119800	*ADCYAP1*-3′UTR	*ADCYAP1*	Brain
X:34866869	*GABRA3*-Non_Synonymous	*GABRA3*	Brain
X:40313487	*FLNA*-Intron	*FLNA*	All tissues except heart and liver

### Validation of A-to-I editing sites by EST sequences

In order to further confirm that the identified A-to-I editing sites represented bona fide RNA editing events rather than technical artifacts, we looked for evidence of editing sites in the public bovine EST database by BLAST alignment. We found that 66% (458/697) of the A-to-I edited sites identified in this study were corroborated in at least one EST clone and 269 sites were supported by more than five edited EST sequences (S3 File). We also investigated the C-to-U and non-canonical editing sites by EST sequences. Results showed that 14 of 21 C-to-U (~66%) were supported by at least one EST clone. However, only 27 of 76 (~0.35%) of the non-canonical sites were confirmed, indicating that these may be considered as false positives.

### Clustered editing sites

A large number of A-to-I editing sites are often clustered together due to promiscuous simultaneous editing of multiple adenosines by *ADAR* proteins [21]. Here, we found that most of the identified editing sites were spatially isolated from each other and only 241 editing sites were detected in 105 clusters (≥2 editing sites within a 100 bp window) which corresponded to 67 known genes (S3 File). Clustered edited sites could be considered as an appropriate feature to distinguish true editing sites from false positive sites [46]. Therefore, we extended the editing sites based on the fact that A-to-I editing sites tend to be clustered together within specific genomic regions. To do this, all of the SNVs that remained after applying the GATK standard filters and removing known SNPs in all the 27 samples were used for extending the editing sites. Then, any SNV within 100 bp from a high-confidence A-to-I RNA editing site (initial candidate editing sites) were added to the pool of editing sites. Approximately 97% of the SNVs identified using this method (903 of 931 SNVs) were A-to-I substitutions and the number of A-to-I RNA editing sites was increased to 1,600. By extending the editing sites with distances smaller than 100 bp surrounding the high-confidence A-to-I editing sites, we identified a total of 1,424 editing sites in 373 clusters and in 223 annotated genes with four editing sites on each cluster on average, as well as 176 editing sites at single sites. We also found 14 edited genes with more than 10 editing sites.

We next assessed the different characteristics of the novel A-to-I editing sites. Results showed that these editing events shared the same characteristics with the high-confidence A-to-I RNA editing site and were less likely to result from sequencing errors. Distribution of the novel A-to-I editing sites in different tissues were 318, 202, 44, 273, 335, 251, 7, 207 and 64 in brain, colon, heart, kidney, liver, lung, skeletal muscle, spleen and testes, respectively, which were similar to high-confidence editing sites. EST analysis revealed that 649 (~72%) and 417 (~46%) of 903 new editing sites were supported by at least one and more than five edited EST sequences, respectively. Similar to known RNA editing sites, we observed a sequence preference for strong G depletion and enrichment in 5′ and 3′ of the new editing sites, respectively. In addition, distribution of the new editing sites in genomic regions revealed that, like high-confidence editing sites, most of these sites were located in non-coding regions (57 sites in upstream, 5 sites in CDSs, 281 sites in intron, 21 sites in 3′ UTR, 327 sites in downstream, 211 sites in intergenic and one site in pseudogene). Our results show that the clusters were largely from inverted repeats regions (329 clusters), however, we found 44 editing clusters from non-repetitive regions. Also, of the 329 clusters in inverted repeats regions, 307 clusters were found in SINE repetitive regions. Thus, these features demonstrated the applicability of this approach in calling new extended RNA editing sites. Detailed characteristics of the new A-to-I editing sites along with high-confidence editing sites are provided in S7 File.

## Discussion

RNA editing increases the proteome and transcriptome diversity in eukaryotic genomes [8, 12, 46, 47]. While in human [8, 12, 14] and mouse [16], the A-to-I RNA editome is well characterized, in bovine only two studies have reported on this type of RNA posttranscriptional modification [26, 27]. Hence, the comprehensive identification of the bovine editome is needed for the understanding of post-transcriptional gene regulation in this agriculturally important species.

The accurate identification of the editome solely from transcriptomic data is technically challenging and may suffer from methodical artifacts such as mapping and sequencing errors [48]. However, recent studies have showed that using efficient computational methods along with appropriate filtering can significantly improve the accuracy of identifying the RNA editing events [4, 49, 50]. Here, we used a RNA-Seq based computational approach for the de novo identification of RNA editing in the absence of the related DNA information. Similar approaches based on only RNA-Seq datasets have been used to characterize human and drosophila editing sites in various tissues [20, 21]. Our approach involved three main steps: alignment of reads to the reference bovine genome, SNVs identification with basic filters, and RNA editing discovery with more stringent filters. On the first step, we made an improvement in the alignment procedure using an accurate aligner and a series of stringent filters. This rigorous filtering strategy mitigate potentially ambiguous mappings to similar genomic regions and mapping bias due to sequencing errors. For the next two steps, a series of filters with stringent cutoffs were applied to reduce obvious sources of false positives such a sequencing/mapping errors, known genomic SNPs and misaligned reads. In present study, beyond the comprehensive filters previously applied to remove potentially erroneous editing events, we used an additional filter and kept the SNVs, which were observed in at least two biological replicates. We hypothesized that functionally relevant RNA editing sites tend to be recurrently edited across individuals, whereas SNPs or false positives are unlikely to be common to unrelated individuals. The usefulness of considering biological replicates to identify biologically meaningful RNA editing sites, has been highlighted in a previous study [18]. Furthermore, we used strand-specific RNA-Seq data, which enable us to characterize the strand of the identified variants and minimize the false positive results [46]. Taken together, as we discussed above, these activities ensured the discovery of an accurate list of editing events in bovine.

The two canonical RNA editing events namely A-to-I and C-to-U together accounted for 91% of the candidate editing sites in our study. Consistent with previous studies, about 88% (697) of these sites corresponded to A-to-I differences, mediated by *ADAR* enzymes, including 555 changes not previously reported in bovine. This finding supported the existing knowledge that A-to-I RNA editing is the dominant type of RNA editing in mammals [4]. Comparison of our results before and after filtering processes showed a clear enrichment in canonical A-to-I editing sites throughout successive filters, which is an indication of the reliability of our approach. Notably, non-canonical changes have been found by previous studies to be the most common sequencing error [51]. Consistently, our EST analysis also confirmed that the non-canonical mismatches might be false positive as only 35% of non-canonical sites were validated by EST sequences. In addition to A-to-I editing sites, about 3% of the identified DNA–RNA differences (21 sites) were C-to-U editing events, which has also been documented in mammals [15, 22]. This type of modification had not yet been reported in cattle. Identification of this type of RNA editing in our datasets can be assigned to the activity of members of the *APOBEC* enzyme family [22].

In the present study, to reduce false positive results and to obtain a high-confidence set of A-to-I editing sites, we used a stringent approach along with a series of filters with stringent cutoffs. However, incorporation of such filters often limits the results and it is undeniable that some of the true editing sites could be removed. Moreover, we focused on editing sites which were edited in at least two biological repeats by using a rigorous filtering approach, it would cause the omission of some true editing sites. For example, one of the applied filters was removing SNVs with very high or low editing ratio, which in addition to removing false positive sites can be lead to filter out some true editing sites. In addition, we used RNA-Seq samples with different coverage, which led us to identify a larger number of SNVs in high coverage samples than in the moderate coverage ones, a finding in agreement with previous studies [22, 46]. It is well known that the number of RNA editing sites depends markedly on sequencing depth and increase with additional reads [22, 46]. Hence, a large portion of this variability could be due to differences in the sequence depth of each sample rather than biological differences between cattle. Therefore, it can be suggested that many sites are edited with low editing levels and sufficient sequencing depth is required to detect these edited sites. In fact, most of the potential editing sites in the high coverage samples were discarded due to lack of supportive evidence from the two other moderate coverage samples. Therefore, we should note that there are likely many more RNA editing sites than those identified by our method. For example, the extent of the clustered A-to-I edited sites for our data set (697 sites) were considerably lower than what has been found in C. elegans and human [6, 21, 52].

We successfully applied a method to extend the identified RNA editing sites. By this method, which queried 100 bp of flanking region, an additional 903 A-to-I editing sites were discovered, including 791 novel ones. Notably, features of the editing sites called by the extending method were the same to those from our multifilter pipeline (high-confidence editing sites). Overall, we identified 1,725 variants (794 variants by the first method and 931 variants by the extending method) of which 1600 (697 sites from the first method and 903 sites by the extending method) and 25 variants (21 sites from the first method and 4 sites by the extending method) were A-to-I and C-to-U editing sites candidates, respectively. Therefore, if we assume that all the variations except A-to-I and C-to-U reflect sequencing errors, then the false discovery rate in this study is ~6%, in agreement with the recent studies that have demonstrated that the non-canonical editing sites are false positives [51]. These noncanonical variants are most likely to be derived from sequencing or mapping errors as well as undetected SNPs in the bovine genome. Also, results of extending the editing sites revealed that most of predicted A-to-I editing sites were ain clusters and were enriched in repetitive regions. The extent of clustered edited sites after extending the editing sites (~89% of 1600 editing sites) is consistent with what is reported in the DARNED database (85.02%) [47]. Therefore, our results revealed that the RNA editing sites tended to reside in clusters instead of being randomly distributed over the genes.

A-to-I editing sites identified in this study yielded a total of 1,338, 86 and 176 editing sites in SINE regions, repetitive non-SINE regions and non-repetitive regions, respectively, an expansion in the list of bovine RNA editing sites. These findings are completely in agreement with known properties of known RNA editing sites and suggests that promiscuous A-to-I editing events can possibly occur in non-repetitive regions [21]. The bovine genome does not have *Alu* repeats, however, there are ral related active SINEs such as Bov-A2 and *Bov-tA* family, which are well known in bovine [53]. Similar to findings in human where most of the A-to-I editing sites are clustered in *Alu* elements [4, 13], our results show that editing sites were enriched in *Bov-tA* family of SINEs (1,069 of 1,338 editing sites where located in SINE regions). On the other hand, nearly all of the editing sites (~99%) targeted non-coding regions (introns, intergenics or untranslated region), similar to what has been documented for human [4]. Also, it is reported that both intronic and non-coding sequences exhibit secondary structure, which can potentially enable the activity of RNA editing enzymes [54]. Moreover, coding regions have fewer inverted repeat sequences, which explains why RNA editing sites mainly reside in non-coding regions [4]. Thus, our findings support the notion that *ADAR* enzymes’ activity is unspecifically influenced by dsRNA [18].

In line with previous studies, a large number of editing sites in introns (478 of 1600, ~30%) and in 3′ UTR or downstream regions (602 of 1600, ~38%) indicates that RNA editing may play fundamental roles in regulation of splicing and miRNA regulation, respectively [12, 55]. Human genome-wide projects, such as ENCODE (Encyclopedia of DNA Elements), have clearly revealed that a considerable fraction of genome are transcribed [56]. Thus, identifying RNA editing sites in intergenic regions may be due to an incomplete or poorly annotated bovine genome. On the other hand, three of the editing sites occurred in known pseudogenes. RNA editing in pseudogenes has been already reported in human tissues [52]. Recently it has been revealed that pseudogenes are functional and can be transcribed. Moreover, they can regulate gene expression by different mechanismes such as miRNA sponge [57, 58]. Hence, in accordance with previous study [52], it can be hypothesizes that RNA editing may be an important mechanism for gene expression regulation by pseudogenes.

The results of this study indicate that the function of the edited genes with amino acid recoding potential are associated with the sepcific function of the tissue. For example, functional analysis based on the nine edited genes in brain revealed that these genes are involved in biological processes such as “chemical synaptic transmission”, “regulation of synaptic plasticity” and “modulation of synaptic transmission”, which is consistent with the known functions of RNA editing in animal nervous systems [59]. We also found that *COG3* is edited in colon, heart, kidney, liver, lung, spleen and testes but not in brain and skeletal muscle. This RNA editing resulted in a codon change from AUU to GUU, and an amino acid recoding from isoleucine to valine. This gene is one of eight subunits of the *COG* tethering complex, which have an important cellular function associated with the structure and function of the Golgi. Interstingly, RNA editing in *COG3* is conserved in human, mouse, rat [60], bovine [26], pig [55] and chicken [61].

Our results show that the number of editing sites are different among tissues, which can be ascribed to tissue-specific roles of RNA editing and sequencing depth variation. The fewer number of identified editing sites in skeletal muscle compared to other tissues suggests lower RNA editing activity in skeletal muscle and is in agreement with previous studies in pig [55] and rhesus macaque [50]. Furthermore, after skeletal muscle, the heart showed the lowest number of A-to-I editing sites (76 sites) which is in accordance to what has been observed in humans [52]. Notably, annotation of the edited genes in each tissue uncovered that most of them were associated with the function of that tissue. For instance, one of the most highly edited genes in brain was *ATCAY*, with seven A-to-I editing sites in its downstream (or its extended 3′ UTR) region. In addition, this gene was edited only in brain tissue. Previous studies show that ATCAY is preferentially expressed in the human brain, is a brain-specific member of the BNIP-2 family and encodes the brain-specific protein *BNIP-H* or *Caytaxin* [62]. This gene affect glutamate synthesis at synapses during neurotransmission by decreasing the steady-state levels of glutamate [63]. Hence, extensive RNA editing of this gene may reflect a functional need for fine-tuning gene expression associated with brain functions. Another gene with a high number of editing sites compared to other genes was *EIF2AK2*. *EIF2AK2* encodes the dsRNA-activated protein kinase R (*PKR*), which is an integral component of the innate immune response and also plays a more general role in cellular physiology such as regulation of protein synthesis, cell proliferation and differentiation, signal transduction and apoptosis [64]. It is also reported that this gene is ubiquitously expressed in all cells at low abundance [65]. Consistently, in our study, *EIF2AK2* was edited in all tissues (except heart and skeletal muscle). This editing ranged from seven sites in lung to one site in testes. Therefore, ubiquitous editing of this gene may represent a functional mechanism for post-transcriptional regulation of this gene.

Our findings of tissue specificity of editing sites are in consistent with previous studies as reported that RNA editing profiles are highly tissue-specific [52, 66]. Brain showed the highest number of tissue-specific edited genes which is consistent with what has been documented for mammals [4, 52]. In addition, existence of tissue-specific RNA editing in other tissues (such as liver) suggests that RNA editing likely plays important roles in non-brain tissues. There are two main hypotheses to explain differential or tissue-specific RNA editing. The first one states that there is a difference in the expression levels of the transcript being edited. In other words, the transcript being edited in one tissue, is not available or at sufficient abundance to be edited in another tissue. To investigate this hypothesis, we assessed the expression of tissue-specific edited genes. In line with a previous study [52], our findings showed that RNA editing pattern and tissue-specific editing are not a consequence of the tissue-specific expression of the edited genes. A second hypothesis is that the editing patterns observed in our samples are related to the expression levels observed for the corresponding *ADAR* enzymes. Positive correlation between ADARs enzymes expression and RNA editing levels in mammalian tissues is reported in previous studies [50, 52]. Deaminase activity of *ADAR* and *ADARB1* and their highest expression in the brain have been demonstrated in previous studies [67]. Here, our results showed a positive correlation between *ADAR* expression and editing levels in different tissues. Nevertheless, even though transcripts in the liver and brain had a large number edited sites, the transcripts of the *ADAR* proteins were not as abundant in the liver as they were in the brain. Hence, biologically it is possible that there are some unknown mechanisms, which mediates the editing levels in different tissues which has also been suggested by others [67, 68].

In bovine, like other mammals, the *ADAR* family is composed of three independent genes, *ADAR* (or *ADAR1*), *ADARB1* (or *ADAR2*), and *ADARB2* (or *ADAR3*) [69]. In our study, *ADARB1* showed highest expression in brain and lung and a very low expression in other tissues. Interestingly, high expression of *ADARB1* in brain and lung has been reported in human [52] and rhesus macaque tissues [50]. It is well known that *ADARB2* is a brain specific enzyme with unknown catalytic activity [52]. In this context, our results also confirmed that *ADARB2* was expressed exclusively in brain, which indicates conserved expression patterns of these genes. Generally, our results suggest that instead of gene expression of edited genes, editing enzyme expression may play an essential role in regulating tissue-specific editing levels in bovine.

Here, *ADAR1* enzyme preferentially targets adenosines when the 5′ nearest neighbor is A ≈ U > C > G, a finding in agreement with the previous study in human [21]. Further, previous studies have reported that a guanosine directly adjacent in the 3′ and a uridine directly adjacent in the 5′ position of an adenosine increase the chance of the adenosine being edited as a potential cis-regulatory mechanism [4], which is in accordance to our findings and may be functional in the formation of a proper *ADAR* substrate structure [70].

We found 16 conserved A-to-I editing sites in 12 protein coding genes. These genes represent the same human orthologous genes and are significantly enriched in the functional category of “chemical synaptic transmission”, which is associated with neural system functions. Of note, seven of the 12 genes were edited in brain and nine of them have amino acid recoding potential, a finding in accordance to others [71]. A-to-I editing of all of these genes except *GIPC1*, *DDX6* and *ADCYAP1* have been reported as conserved between human and mouse [71]. Most of the conserved edited genes were associated with neural-specific functions [71]. The observed low overlap between editing sites identified in this study and human editome is not surprising, as it is well documented that only very few editing sites are known to be conserved across large evolutionary distances [71, 72] [66, 71].

We compared our results to RNA editing sites predicted by Chen et al. [26]. This comparison revealed 254 common A-to-I editing sites, which reside in 91 different genes. Five of the 91 genes (including *GABRA3*, *GRIK2*, *COG3*, *CADPS* and *CYFIP2*) have editing sites with non-synonymous shifts. Thus, we report six novel putative functional coding changing editing sites in the genes *AZIN1*, *GIPC1*, *KCNA1*, *LEMD2*, *TMEM63B* and *SON* in bovine transcriptome. Five of these genes have been reported as genes with functional coding changing editing sites in mammals species including *KCNA1* in brain tissue of mouse [73]; *TMEM63B*, *AZIN1* and *SON* in human [8, 74] and mouse [16] and *GIPC1* in human [75], thus further demonstrating the efficacy of our A-to-I prediction pipelines and reveals that some edited sites are conserved throughout mammalian evolution. Among the novel edited genes with non-synonymous changes, only five genes (including *CADPS*, *CYFIP2*, *GRIK2*, *KCNA1* and *TMEM63B*) showed tissue-specific editing. A certain proportion of the observed differences in the editing patterns between our results and [26] could be attributed to variability in number and age of tissues, computational method for editing discovery and differences in the stringency of applied filters, differences in experimental design and variability in the sequence depth of samples. This also might indicate diversity of editome among tissues or individuals. Overall, we discovered 1,346 novel A-to-I editing sites not yet reported in bovine transcriptome.

## Conclusion

Here, we used a large number of RNA-Seq samples along with a computational method with multiple filters and stringent thresholds to facilitate unbiased detection of bona fide RNA editing sites in the bovine genome in the absence of corresponding DNA information. The present study extends the list of RNA editing sites in bovine and provides pipelines that may be used to investigate the editome in other organisms.

## Supporting information

S1 File Summary statistics for reads and alignment information of different samples. (XLS)

S2 File Final list of all identified DNA-RNA differences in different tissues of bovine. (XLSX)

S3 File Features of all the identified A-to-I editing sites in bovine transcriptome. (XLSX)

S4 File List of tissue-specific edited genes in different tissues of bovine and gene enrichment analysis of these genes. Also, editing ratio of each editing site in each tissue is provided.
(XLSX)

S5 File List of all edited genes (A-to-I editing) with editing number in different tissues in bovine. (XLSX)

S6 File Gene expression of tissues-specific edited genes in different tissues. (XLSX)

S7 File List of all A-to-I editing (high-confidence and extended) in different tissues in bovine. (XLSX)

## References

[1] Djebali S, Davis CA, Merkel A, Dobin A, Lassmann T, Mortazavi A, et al Landscape of transcription in human cells. Nature. 2012;489(7414):101–8. doi: 10.1038/nature11233 22955620 10.1038/nature11233 PMC3684276

[2] Derrien T, Johnson R, Bussotti G, Tanzer A, Djebali S, Tilgner H, et al The GENCODE v7 catalog of human long noncoding RNAs: analysis of their gene structure, evolution, and expression. Genome research. 2012;22(9):1775–89. doi: 10.1101/gr.132159.111 22955988 10.1101/gr.132159.111 PMC3431493

[3] Consortium EP. An integrated encyclopedia of DNA elements in the human genome. Nature. 2012;489(7414):57–74. doi: 10.1038/nature11247 22955616 10.1038/nature11247 PMC3439153

[4] Nishikura K. A-to-I editing of coding and non-coding RNAs by ADARs. Nature Reviews Molecular Cell Biology. 2015. 10.1038/nrm.2015.4 PMC4824625 26648264

[5] Benne R, Van Den Burg J, Brakenhoff JP, Sloof P, Van Boom JH, Tromp MC. Major transcript of the frameshifted coxll gene from trypanosome mitochondria contains four nucleotides that are not encoded in the DNA. Cell. 1986;46(6):819–26. 3019552 10.1016/0092-8674(86)90063-2

[6] Zhao H-Q, Zhang P, Gao H, He X, Dou Y, Huang AY, et al Profiling the RNA editomes of wild-type C. elegans and ADAR mutants. Genome research. 2015;25(1):66–75. doi: 10.1101/gr.176107.114 25373143 10.1101/gr.176107.114 PMC4317174

[7] Martínez‐Montes A, Fernandez A, Pérez‐Montarelo D, Alves E, Benitez R, Nunez Y, et al Using RNA‐Seq SNP data to reveal potential causal mutations related to pig production traits and RNA editing. Animal Genetics. 2017;48(2):151–65. doi: 10.1111/age.12507 27642173 10.1111/age.12507

[8] Peng Z, Cheng Y, Tan BC-M, Kang L, Tian Z, Zhu Y, et al Comprehensive analysis of RNA-Seq data reveals extensive RNA editing in a human transcriptome. Nature biotechnology. 2012;30(3):253–60. doi: 10.1038/nbt.2122 22327324 10.1038/nbt.2122

[9] Nishikura K. Functions and regulation of RNA editing by ADAR deaminases. Annual review of biochemistry. 2010;79:321–49. doi: 10.1146/annurev-biochem-060208-105251 20192758 10.1146/annurev-biochem-060208-105251 PMC2953425

[10] Rueter SM, Dawson TR, Emeson RB. Regulation of alternative splicing by RNA editing. Nature. 1999;399(6731):75–80. doi: 10.1038/19992 10331393 10.1038/19992

[11] Heale BS, Keegan LP, O’Connell MA. The effect of RNA editing and ADARs on miRNA biogenesis and function. Regulation of microRNAs: Springer; 2010 p. 76–84. 21627032

[12] Hwang T, Park C-K, Leung AK, Gao Y, Hyde TM, Kleinman JE, et al Dynamic regulation of RNA editing in human brain development and disease. Nature Neuroscience. 2016. 10.1038/nn.4337 27348216

[13] Maas S, Kawahara Y, Tamburro KM, Nishikura K. A-to-I RNA editing and human disease. RNA biology. 2006;3(1):1–9. 17114938 10.4161/rna.3.1.2495 PMC2947206

[14] Han L, Diao L, Yu S, Xu X, Li J, Zhang R, et al The genomic landscape and clinical relevance of A-to-I RNA editing in human cancers. Cancer cell. 2015;28(4):515–28. doi: 10.1016/j.ccell.2015.08.013 26439496 10.1016/j.ccell.2015.08.013 PMC4605878

[15] Rosenberg BR, Hamilton CE, Mwangi MM, Dewell S, Papavasiliou FN. Transcriptome-wide sequencing reveals numerous APOBEC1 mRNA-editing targets in transcript 3′ UTRs. Nature structural & molecular biology. 2011;18(2):230–6. 10.1038/nsmb.1975 PMC3075553 21258325

[16] Danecek P, Nellaker C, McIntyre RE, Buendia-Buendia JE, Bumpstead S, Ponting CP, et al High levels of RNA-editing site conservation amongst 15 laboratory mouse strains. Genome Biol. 2012;13(4):26 doi: 10.1186/gb-2012-13-4-r26 ; PubMed Central PMCID: PMC3446300. 22524474 10.1186/gb-2012-13-4-r26 PMC3446300

[17] Martínez‐Montes A, Fernandez A, Pérez‐Montarelo D, Alves E, Benitez R, Nunez Y, et al Using RNA‐Seq SNP data to reveal potential causal mutations related to pig production traits and RNA editing. Animal Genetics. 2016. 10.1111/age.12507 27642173

[18] Roux P-F, Frésard L, Boutin M, Leroux S, Klopp C, Djari A, et al The Extent of mRNA Editing Is Limited in Chicken Liver and Adipose, but Impacted by Tissular Context, Genotype, Age, and Feeding as Exemplified with a Conserved Edited Site in COG3. G3: Genes| Genomes| Genetics. 2016;6(2):321–35. 10.1534/g3.115.022251 PMC4751552 26637431

[19] Moran B, Butler ST, Creevey CJ. Comparison and Characterisation of Mutation Calling from Whole Exome and RNA Sequencing Data for Liver and Muscle Tissue in Lactating Holstein Cows Divergent for Fertility. bioRxiv. 2017:101733.

[20] Ramaswami G, Zhang R, Piskol R, Keegan LP, Deng P, O'connell MA, et al Identifying RNA editing sites using RNA sequencing data alone. Nature methods. 2013;10(2):128–32. doi: 10.1038/nmeth.2330 23291724 10.1038/nmeth.2330 PMC3676881

[21] Zhu S, Xiang J-F, Chen T, Chen L-L, Yang L. Prediction of constitutive A-to-I editing sites from human transcriptomes in the absence of genomic sequences. BMC genomics. 2013;14(1):206. 23537002 10.1186/1471-2164-14-206 PMC3637798

[22] Blanc V, Park E, Schaefer S, Miller M, Lin Y, Kennedy S, et al Genome-wide identification and functional analysis of Apobec-1-mediated C-to-U RNA editing in mouse small intestine and liver. Genome biology. 2014;15(6):R79 doi: 10.1186/gb-2014-15-6-r79 24946870 10.1186/gb-2014-15-6-r79 PMC4197816

[23] Danecek P, Nellåker C, McIntyre RE, Buendia-Buendia JE, Bumpstead S, Ponting CP, et al High levels of RNA-editing site conservation amongst 15 laboratory mouse strains. Genome biology. 2012;13(4):r26. 10.1186/gb-2012-13-4-r26 PMC3446300 22524474

[24] Gu T, Buaas FW, Simons AK, Ackert-Bicknell CL, Braun RE, Hibbs MA. Canonical A-to-I and C-to-U RNA editing is enriched at 3′ UTRs and microRNA target sites in multiple mouse tissues. PloS one. 2012;7(3):e33720 doi: 10.1371/journal.pone.0033720 22448268 10.1371/journal.pone.0033720 PMC3308996

[25] Lagarrigue S, Hormozdiari F, Martin LJ, Lecerf F, Hasin Y, Rau C, et al Limited RNA editing in exons of mouse liver and adipose. Genetics. 2013;193(4):1107–15. doi: 10.1534/genetics.112.149054 23410828 10.1534/genetics.112.149054 PMC3606090

[26] Chen Z, Hagen DE, Wang J, Elsik CG, Ji T, Siqueira LG, et al Global assessment of imprinted gene expression in the bovine conceptus by next generation sequencing. Epigenetics. 2016;11(7):501–16. doi: 10.1080/15592294.2016.1184805 27245094 10.1080/15592294.2016.1184805 PMC4939914

[27] Porath HT, Knisbacher BA, Eisenberg E, Levanon EY. Massive A-to-I RNA editing is common across the Metazoa and correlates with dsRNA abundance. Genome Biology. 2017;18(1):185 doi: 10.1186/s13059-017-1315-y 28969707 10.1186/s13059-017-1315-y PMC5625713

[28] He T, Lei W, Ge C, Du P, Wang L, Li F. Large-scale detection and analysis of adenosine-to-inosine RNA editing during development in Plutella xylostella. Molecular Genetics and Genomics. 2015;290(3):929–37. doi: 10.1007/s00438-014-0968-4 25492222 10.1007/s00438-014-0968-4

[29] Mazloomian A, Meyer IM. Genome-wide identification and characterization of tissue-specific RNA editing events in D. melanogaster and their potential role in regulating alternative splicing. RNA biology. 2015;12(12):1391–401. doi: 10.1080/15476286.2015.1107703 26512413 10.1080/15476286.2015.1107703 PMC4829317

[30] Merkin J, Russell C, Chen P, Burge CB. Evolutionary dynamics of gene and isoform regulation in Mammalian tissues. Science. 2012;338(6114):1593–9. doi: 10.1126/science.1228186 23258891 10.1126/science.1228186 PMC3568499

[31] Bolger AM, Lohse M, Usadel B. Trimmomatic: a flexible trimmer for Illumina sequence data. Bioinformatics. 2014;30(15):2114–20. doi: 10.1093/bioinformatics/btu170 24695404 10.1093/bioinformatics/btu170 PMC4103590

[32] Kim D, Langmead B, Salzberg SL. HISAT: a fast spliced aligner with low memory requirements. Nature methods. 2015;12(4):357–60. doi: 10.1038/nmeth.3317 25751142 10.1038/nmeth.3317 PMC4655817

[33] Xiong H, Liu D, Li Q, Lei M, Xu L, Wu L, et al RED-ML: a novel, effective RNA editing detection method based on machine learning. GigaScience. 2017. 10.1093/gigascience/gix012 PMC5467039 28328004

[34] Piskol R, Ramaswami G, Li JB. Reliable identification of genomic variants from RNA-seq data. The American Journal of Human Genetics. 2013;93(4):641–51. doi: 10.1016/j.ajhg.2013.08.008 24075185 10.1016/j.ajhg.2013.08.008 PMC3791257

[35] DePristo MA, Banks E, Poplin R, Garimella KV, Maguire JR, Hartl C, et al A framework for variation discovery and genotyping using next-generation DNA sequencing data. Nature genetics. 2011;43(5):491–8. doi: 10.1038/ng.806 21478889 10.1038/ng.806 PMC3083463

[36] De Summa S, Malerba G, Pinto R, Mori A, Mijatovic V, Tommasi S. GATK hard filtering: tunable parameters to improve variant calling for next generation sequencing targeted gene panel data. BMC bioinformatics. 2017;18(Suppl 5):119 doi: 10.1186/s12859-017-1537-8 ; PubMed Central PMCID: PMC5374681. 28361668 10.1186/s12859-017-1537-8 PMC5374681

[37] Wang X, Lu P, Luo Z. GMATo: a novel tool for the identification and analysis of microsatellites in large genomes. Bioinformation. 2013;9(10):541–4. doi: 10.6026/97320630009541 23861572 10.6026/97320630009541 PMC3705631

[38] Kent WJ. BLAT—the BLAST-like alignment tool. Genome research. 2002;12(4):656–64. doi: 10.1101/gr.229202 11932250 10.1101/gr.229202 PMC187518

[39] Smit AF, Hubley R, Green P. RepeatMasker. 1996.

[40] Cingolani P. snpEff: Variant effect prediction. 2012.

[41] Chen EY, Tan CM, Kou Y, Duan Q, Wang Z, Meirelles GV, et al Enrichr: interactive and collaborative HTML5 gene list enrichment analysis tool. BMC bioinformatics. 2013;14(1):128. 23586463 10.1186/1471-2105-14-128 PMC3637064

[42] Crooks GE, Hon G, Chandonia J-M, Brenner SE. WebLogo: a sequence logo generator. Genome research. 2004;14(6):1188–90. doi: 10.1101/gr.849004 15173120 10.1101/gr.849004 PMC419797

[43] Patro R, Duggal G, Love MI, Irizarry RA, Kingsford C. Salmon provides fast and bias-aware quantification of transcript expression. Nat Methods. 2017;14(4):417–9. doi: 10.1038/nmeth.4197 . 28263959 10.1038/nmeth.4197 PMC5600148

[44] Ramaswami G, Li JB. Identification of human RNA editing sites: A historical perspective. Methods. 2016;107:42–7. doi: 10.1016/j.ymeth.2016.05.011 27208508 10.1016/j.ymeth.2016.05.011 PMC5014717

[45] Li Q, Wang Z, Lian J, Schiøtt M, Jin L, Zhang P, et al Caste-specific RNA editomes in the leaf-cutting ant Acromyrmex echinatior. Nature communications. 2014;5. 10.1038/ncomms5943 PMC4200514 25266559

[46] Bazak L, Haviv A, Barak M, Jacob-Hirsch J, Deng P, Zhang R, et al A-to-I RNA editing occurs at over a hundred million genomic sites, located in a majority of human genes. Genome research. 2014;24(3):365–76. doi: 10.1101/gr.164749.113 24347612 10.1101/gr.164749.113 PMC3941102

[47] Kiran A, Baranov PV. DARNED: a DAtabase of RNa EDiting in humans. Bioinformatics. 2010;26(14):1772–6. doi: 10.1093/bioinformatics/btq285 20547637 10.1093/bioinformatics/btq285

[48] Wang Z, Lian J, Li Q, Zhang P, Zhou Y, Zhan X, et al RES-Scanner: a software package for genome-wide identification of RNA-editing sites. GigaScience. 2016;5(1):37 doi: 10.1186/s13742-016-0143-4 27538485 10.1186/s13742-016-0143-4 PMC4989487

[49] Ramaswami G, Lin W, Piskol R, Tan MH, Davis C, Li JB. Accurate identification of human Alu and non-Alu RNA editing sites. Nature methods. 2012;9(6):579–81. doi: 10.1038/nmeth.1982 22484847 10.1038/nmeth.1982 PMC3662811

[50] Chen J-Y, Peng Z, Zhang R, Yang X-Z, Tan BC-M, Fang H, et al RNA editome in rhesus macaque shaped by purifying selection. PLoS genet. 2014;10(4):e1004274 doi: 10.1371/journal.pgen.1004274 24722121 10.1371/journal.pgen.1004274 PMC3983040

[51] Piskol R, Peng Z, Wang J, Li JB. Lack of evidence for existence of noncanonical RNA editing. Nature biotechnology. 2013;31(1):19–20. doi: 10.1038/nbt.2472 23302925 10.1038/nbt.2472

[52] Picardi E, Manzari C, Mastropasqua F, Aiello I, D’Erchia AM, Pesole G. Profiling RNA editing in human tissues: towards the inosinome Atlas. Scientific reports. 2015;5. 10.1038/srep14941 PMC4598827 26449202

[53] Adelson DL, Raison JM, Edgar RC. Characterization and distribution of retrotransposons and simple sequence repeats in the bovine genome. Proceedings of the National Academy of Sciences. 2009;106(31):12855–60. 10.1073/pnas.0901282106 PMC2722308 19625614

[54] Washietl S, Hofacker IL, Lukasser M, Hüttenhofer A, Stadler PF. Mapping of conserved RNA secondary structures predicts thousands of functional noncoding RNAs in the human genome. Nature biotechnology. 2005;23(11):1383–90. doi: 10.1038/nbt1144 16273071 10.1038/nbt1144

[55] Funkhouser SA, Steibel JP, Bates RO, Raney NE, Schenk D, Ernst CW. Evidence for Transcriptome-wide RNA Editing Among Sus scrofa PRE-1 SINE Elements. bioRxiv. 2017:096321. 10.1186/s12864-017-3766-7 PMC5423416 28486975

[56] Gunter C, Snyder M. An integrated encyclopedia of DNA elements in the human genome. Nature. 2012;489(7414):5774. 10.1038/nature11247 PMC3439153 22955616

[57] Le TD, Zhang J, Liu L, Li J. Computational methods for identifying miRNA sponge interactions. Briefings in bioinformatics. 2016:bbw042. 10.1093/bib/bbw042 27273287

[58] Goodhead I, Darby AC. Taking the pseudo out of pseudogenes. Current opinion in microbiology. 2015;23:102–9. doi: 10.1016/j.mib.2014.11.012 25461580 10.1016/j.mib.2014.11.012

[59] Jepson JE, Reenan RA. RNA editing in regulating gene expression in the brain. Biochimica et Biophysica Acta (BBA)-Gene Regulatory Mechanisms. 2008;1779(8):459–70. 18086576 10.1016/j.bbagrm.2007.11.009

[60] Holmes AP, Wood SH, Merry BJ, de Magalhaes JP. A-to-I RNA editing does not change with age in the healthy male rat brain. Biogerontology. 2013;14(4):395–400. doi: 10.1007/s10522-013-9433-8 23708854 10.1007/s10522-013-9433-8 PMC3739863

[61] Fresard L, Leroux S, Roux P-F, Klopp C, Fabre S, Esquerre D, et al Genome-Wide Characterization of RNA Editing in Chicken Embryos Reveals Common Features among Vertebrates. PloS one. 2015;10(5):e0126776 doi: 10.1371/journal.pone.0126776 26024316 10.1371/journal.pone.0126776 PMC4449034

[62] Liu X, Zhang G, Zhang C, Wang J. Predicted Trans-Acting siRNAs in the Human Brain. International journal of molecular sciences. 2015;16(2):3377–90. doi: 10.3390/ijms16023377 25654231 10.3390/ijms16023377 PMC4346901

[63] Piro RM, Molineris I, Ala U, Di Cunto F. Evaluation of candidate genes from orphan FEB and GEFS+ loci by analysis of human brain gene expression atlases. PLoS One. 2011;6(8):e23149 doi: 10.1371/journal.pone.0023149 21858011 10.1371/journal.pone.0023149 PMC3157479

[64] Oguejiofor CF, Cheng Z, Abudureyimu A, Anstaett OL, Brownlie J, Fouladi-Nashta AA, et al Global Transcriptomic Profiling of Bovine Endometrial Immune Response In Vitro. II. Effect of Bovine Viral Diarrhea Virus on the Endometrial Response to Lipopolysaccharide 1. Biology of reproduction. 2015;93(4):Article 101, 1–16. 10.1095/biolreprod.115.128876 26353892

[65] Hober D, Sauter P. Pathogenesis of type 1 diabetes mellitus: interplay between enterovirus and host. Nature Reviews Endocrinology. 2010;6(5):279–89. doi: 10.1038/nrendo.2010.27 20351698 10.1038/nrendo.2010.27

[66] Li JB, Levanon EY, Yoon J-K, Aach J, Xie B, LeProust E, et al Genome-wide identification of human RNA editing sites by parallel DNA capturing and sequencing. Science. 2009;324(5931):1210–3. doi: 10.1126/science.1170995 19478186 10.1126/science.1170995

[67] Wahlstedt H, Daniel C, Enstero M, Ohman M. Large-scale mRNA sequencing determines global regulation of RNA editing during brain development. Genome Res. 2009;19(6):978–86. doi: 10.1101/gr.089409.108 ; PubMed Central PMCID: PMC2694479. 19420382 10.1101/gr.089409.108 PMC2694479

[68] Osenberg S, Yaacov NP, Safran M, Moshkovitz S, Shtrichman R, Sherf O, et al Alu sequences in undifferentiated human embryonic stem cells display high levels of A-to-I RNA editing. PLoS one. 2010;5(6):e11173 doi: 10.1371/journal.pone.0011173 20574523 10.1371/journal.pone.0011173 PMC2888580

[69] Chen C-X, CHO D-SC, WANG Q, LAI F, CARTER KC, NISHIKURA K. A third member of the RNA-specific adenosine deaminase gene family, ADAR3, contains both single-and double-stranded RNA binding domains. Rna. 2000;6(5):755–67. 10836796 10.1017/s1355838200000170 PMC1369955

[70] Lehmann KA, Bass BL. Double-stranded RNA adenosine deaminases ADAR1 and ADAR2 have overlapping specificities. Biochemistry. 2000;39(42):12875–84. 11041852 10.1021/bi001383g

[71] Pinto Y, Cohen HY, Levanon EY. Mammalian conserved ADAR targets comprise only a small fragment of the human editosome. Genome biology. 2014;15(1):R5 doi: 10.1186/gb-2014-15-1-r5 24393560 10.1186/gb-2014-15-1-r5 PMC4053846

[72] Reenan RA. Molecular determinants and guided evolution of species-specific RNA editing. Nature. 2005;434(7031):409–13. doi: 10.1038/nature03364 15772668 10.1038/nature03364

[73] Cattenoz PB, Taft RJ, Westhof E, Mattick JS. Transcriptome-wide identification of A> I RNA editing sites by inosine specific cleavage. Rna. 2013;19(2):257–70. doi: 10.1261/rna.036202.112 23264566 10.1261/rna.036202.112 PMC3543087

[74] Mo F, Wyatt AW, Sun Y, Brahmbhatt S, McConeghy BJ, Wu C, et al Systematic identification and characterization of RNA editing in prostate tumors. PloS one. 2014;9(7):e101431 doi: 10.1371/journal.pone.0101431 25036877 10.1371/journal.pone.0101431 PMC4103770

[75] Solomon O, Bazak L, Levanon EY, Amariglio N, Unger R, Rechavi G, et al Characterizing of functional human coding RNA editing from evolutionary, structural, and dynamic perspectives. Proteins: Structure, Function, and Bioinformatics. 2014;82(11):3117–31. 10.1002/prot.24672 25136968

